# Genetic overlap between treatment-resistant schizophrenia and smoking initiation

**DOI:** 10.1093/ijnp/pyag035

**Published:** 2026-06-28

**Authors:** Elise Koch, Nadine Parker, Helin H Mohammad, Hasan Ç Lenk, Lars A R Ystaas, Piotr Jaholkowski, Alexey A Shadrin, Oleksandr Frei, Anders M Dale, Srdjan Djurovic, Espen Molden, Kevin S O’Connell, Ole A Andreassen

**Affiliations:** Centre for Precision Psychiatry, Division of Mental Health and Addiction, Oslo University Hospital, and Institute of Clinical Medicine, University of Oslo, Oslo, Norway; Center for Psychopharmacology, Diakonhjemmet Hospital, Oslo, Norway; Centre for Precision Psychiatry, Division of Mental Health and Addiction, Oslo University Hospital, and Institute of Clinical Medicine, University of Oslo, Oslo, Norway; Department of Clinical and Molecular Medicine, Faculty of Medicine and Health Sciences, Norwegian University of Science and Technology, Trondheim, Norway; Center for Psychopharmacology, Diakonhjemmet Hospital, Oslo, Norway; Centre for Precision Psychiatry, Division of Mental Health and Addiction, Oslo University Hospital, and Institute of Clinical Medicine, University of Oslo, Oslo, Norway; Centre for Precision Psychiatry, Division of Mental Health and Addiction, Oslo University Hospital, and Institute of Clinical Medicine, University of Oslo, Oslo, Norway; Centre for Precision Psychiatry, Division of Mental Health and Addiction, Oslo University Hospital, and Institute of Clinical Medicine, University of Oslo, Oslo, Norway; KG Jebsen Centre for Neurodevelopmental Disorders, University of Oslo and Oslo University Hospital, Oslo, Norway; Centre for Precision Psychiatry, Division of Mental Health and Addiction, Oslo University Hospital, and Institute of Clinical Medicine, University of Oslo, Oslo, Norway; Center for Bioinformatics, Department of Informatics, University of Oslo, 0316, Oslo, Norway; Centre for Precision Psychiatry, Division of Mental Health and Addiction, Oslo University Hospital, and Institute of Clinical Medicine, University of Oslo, Oslo, Norway; Center for Multimodal Imaging and Genetics, J. Craig Venter Institute, La Jolla, CA, United States; Department of Neurosciences, University of California San Diego, La Jolla, CA, United States; Centre for Precision Psychiatry, Division of Mental Health and Addiction, Oslo University Hospital, and Institute of Clinical Medicine, University of Oslo, Oslo, Norway; Department of Medical Genetics, Oslo University Hospital and University of Oslo, Oslo, Norway; Center for Psychopharmacology, Diakonhjemmet Hospital, Oslo, Norway; Centre for Precision Psychiatry, Division of Mental Health and Addiction, Oslo University Hospital, and Institute of Clinical Medicine, University of Oslo, Oslo, Norway; Centre for Precision Psychiatry, Division of Mental Health and Addiction, Oslo University Hospital, and Institute of Clinical Medicine, University of Oslo, Oslo, Norway; KG Jebsen Centre for Neurodevelopmental Disorders, University of Oslo and Oslo University Hospital, Oslo, Norway

**Keywords:** treatment-resistant schizophrenia, smoking, clozapine, genetics, GWAS

## Abstract

**Background:**

Early detection of treatment-resistant schizophrenia (TRS) is of substantial clinical importance. While TRS is heritable, the associated genetic variants have been difficult to identify. Cigarette smoking is associated with non-response to antipsychotics, and smoking behavior and schizophrenia have a shared genetic basis. Thus, TRS may also have a shared genetic basis with smoking behavior.

**Objective:**

Here, we aim to identify genetic variants associated with TRS by leveraging overlapping genetic variants with smoking initiation to increase statistical power.

**Methods:**

We analyzed genome-wide data for TRS and smoking initiation with the conditional/conjunctional false discovery rate (cond/conjFDR) to identify shared loci, and LD score regression to determine genetic correlations. To investigate potential causal effects of shared loci, we performed Mendelian randomization analyses. Shared loci were mapped to genes, which were further investigated for enrichment of drug target genes.

**Results:**

We observed a significant positive genetic correlation between TRS and smoking initiation (r_g_ = 0.47 *P* = .0002). Leveraging the genetic overlap between TRS and smoking initiation, we identified four novel loci jointly associated with TRS and smoking initiation. The condFDR results improved polygenic prediction of TRS. Mendelian randomization indicates putative evidence for a causal effect of genetic liability to TRS on smoking initiation. The functional genetic analyses suggest that alpha-1-adrenergic receptors may be involved in the pathophysiology of TRS and possibly related to the efficacy of clozapine versus other antipsychotic drugs.

**Conclusion:**

In conclusion, our results show that shared genetic mechanisms influence both TRS and smoking behavior, which provide new insights into the biological underpinnings of TRS.

**Significant outcomes**

In the present study, we boosted discovery of genetic variants associated with TRS and identified four novel loci associated with TRS after conditioning on smoking initiation. Genetic correlation analysis showed a positive genetic correlation between TRS and smoking initiation, and Mendelian randomization analyses showed putative evidence for a causal effect of TRS on smoking initiation. In an independent validation sample, a polygenic score (PGS) based on condFDR-ranked single nucleotide polymorphisms (SNPs) showed greater variance explained in TRS compared to the standard TRS PGS. From protein–protein interaction (PPI) network-based analyses and gene-set enrichment analyses (GSEA), we identified alpha-1-adrenergic receptors as potential targets for TRS.

**Limitations**

It should be noted that the cond/conjFDR method does not identify the specific causal variants underlying the overlapping genomic loci, and that the detection of cross-trait enrichment is influenced by the power of the investigated GWAS. ^1^ Although our results highlight how data from low-powered GWASs can still be useful to study genetic architecture and overlap between traits, our results might be influenced by the small sample size of the TRS GWAS. However, despite identifying novel loci for TRS, larger GWASs of TRS are required to better understand the underlying genetics of TRS. In addition, the predictive ability of the PGS remains far from being clinically relevant, and larger GWASs of TRS will also improve SNP weights for polygenic prediction. The phenotyping of the TRS GWAS samples has not recorded comorbid smoking in the schizophrenia cases, and there could potentially be differences in smoking frequency between TRS and non-TRS patients. Therefore, our findings of genetic overlap between TRS and smoking could theoretically be driven by this possibly different smoking frequency in TRS vs non-TRS patients. Moreover, the smoking GWAS samples may include schizophrenia cases, which could suggest that some of the genetic overlap may be due to shared phenotypes. In addition, nicotine and clozapine may have overlapping pharmacological mechanisms of action on acetylcholine receptors, which may be captured in the identified genetic overlap between smoking initiation and TRS defined by clozapine use. There are several considerations when using clozapine as a proxy phenotype for TRS.^2^ The control group (individuals with schizophrenia who never used clozapine) could still include individuals with poor treatment response or resistance, as clozapine is prescribed only to a fraction of patients with non-response to ≥2 antipsychotic trials.^3^ Of note, clozapine prescription rates differ by country,^4^ sex,^5^ and ethnicity.^6^ Another issue is potential pseudo-resistance, where lack of response can result from sub-therapeutic medication levels due to poor adherence or metabolic status.^7^^,^^8^ These limitations of a clozapine-defined TRS phenotype could inflate the observed genetic overlap with smoking initiation. Finally, the individuals included in the GWAS used in our cond/conjFDR analyses were of predominantly European ancestry, which suggests that the findings may not translate to other ancestry groups.

## Introduction

Treatment-resistant schizophrenia (TRS) is defined as a failure of response to at least two antipsychotic drugs administered in adequate dose and duration, and occurs in about 30% of patients with schizophrenia.^9^ TRS has been associated with increased mortality, higher rates of suicide attempts, and longer hospital stays, leading to substantial economic burdens due to healthcare utilization.^10^ The antipsychotic drug clozapine is superior to other antipsychotics in terms of clinical effect and long-term outcomes.^11–13^ However, due to the risk of rare but potentially fatal hematological adverse effects,^13–15^ clozapine’s only indication is TRS. Clozapine treatment is effective in approximately 60% of TRS cases,^16^ and may decrease mortality in schizophrenia.^11^^,^^17^ Thus, early identification of TRS is of substantial clinical importance, but a significant challenge is the high clinical and biological heterogeneity that characterizes TRS.^18^

Genome-wide association studies (GWAS) have identified hundreds of genetic loci harboring risk variants for schizophrenia.^19^ While little progress has been made in identifying genetic associations with pharmacological treatment outcomes in psychiatry,^20^ emerging evidence suggests that TRS may have a genetic component.^3^^,^^21^ The identification of genetic variants associated with TRS is limited by insufficient sample sizes as well as variability in definitions of TRS status.^22^ No genome-wide significant loci have yet been identified in a GWAS of TRS defined based on the use of clozapine, including the world’s largest sample of 30 826 individuals with schizophrenia (N_TRS_ = 10 501 and N_non-TRS_ = 20 325).^3^

The prevalence of cigarette smoking in schizophrenia is estimated to 70%-80%, which is about three times the rate of the general population.^23^ Hundreds of loci have been identified for tobacco use in a GWAS, including over 1 million individuals,^24^ where smoking phenotypes were positively genetically correlated with schizophrenia.^24^ A Mendelian randomization (MR) study showed that genetic liability to cigarette smoking significantly increases the risk of developing schizophrenia.^25^ However, the underlying factors driving the high smoking rates in schizophrenia patients are unclear.^23^ It has been hypothesized that schizophrenia patients smoke to self-medicate or alleviate negative and cognitive symptoms.^26^^,^^27^ Furthermore, cigarette smoking has been associated with non-response to antipsychotics.^28^ In TRS, cigarette smoking has been associated with more severe negative symptoms.^29^ While it is known that smoking induces CYP1A2,^30^ which metabolizes antipsychotic drugs such as clozapine and olanzapine,^31^^,^^32^ cigarette smoking also decreases the efficacy of olanzapine treatment independently of CYP1A2 genotype.^33^ These findings indicate that smoking behavior and non-response to antipsychotics may have a shared genetic basis.

Considering the limited understanding of the underlying genetics of TRS based on the GWAS findings so far, complementary statistical approaches are needed to gain better understanding of the genetic underpinnings of TRS and to explore the genetic relationship between TRS and smoking behavior. Hypothesizing that TRS has a shared genetic basis with smoking behavior, we aim to identify genetic variants associated with TRS by leveraging overlapping genetic associations with smoking initiation to increase power for genetic discovery.

## Methods

### GWAS sample description

We utilized publicly available GWAS summary statistics for TRS, which included 10 501 TRS cases and 20 325 non-TRS patients, with data derived from the CLOZUK and Psychiatric Genomics Consortium (PGC) cohorts.^3^ For smoking initiation (“ever/never smoked regularly”), data were derived from a large-scale GWAS on smoking initiation in a population sample (*N* = 1 232 091).^24^ All GWAS data utilized in this study are from individuals of European ancestry. The Norwegian Institutional Review Board for the South-East Norway Region has evaluated the current protocol and found that no additional institutional review board approval was needed because no individual data were used.

### Conditional/conjunctional FDR and genetic correlation analysis

To boost discovery of genetic variants associated with TRS, we applied the conditional false discovery rate (condFDR) approach,^34^^,^^35^ using default settings (Supplementary Methods S1). The condFDR re-ranks genetic variants compared to *P*-value-based ranking and increases the power to discover loci associated with a primary phenotype (TRS) by leveraging associations with a secondary phenotype (smoking initiation).^1^^,^^34^^,^^35^ We then performed conjunctional FDR (conjFDR) analyses to identify shared variants between TRS and smoking initiation. In conjFDR, the process of the condFDR analysis is repeated, switching the roles of the primary and secondary phenotypes. The largest condFDR value between the two runs is then used as the conjFDR value. A variant with a conjFDR less than 0.05 was considered as a shared variant (corresponding to 5 false positive per 100 reported associations). To estimate bivariate genetic correlations between TRS and smoking initiation, we utilized linkage disequilibrium score regression (LDSC).^36^ To account for the possible effect of schizophrenia, we have performed multi-trait conditional and joint analysis (mtCOJO).^37^ We conditioned the effect of SNPs estimated for TRS on those of schizophrenia, using summary statistics of a GWAS on schizophrenia including 76 755 cases and 243 649 controls, performed by the PGC.^19^ We then performed genetic correlation analysis between TRS conditioned on schizophrenia and smoking initiation.

### Locus definition, functional annotation and gene mapping

To define genetic loci based on the association summary statistics produced with conjFDR, we used functional mapping and annotation of GWAS (FUMA)^38^ with default settings. Using FUMA,^38^ each lead SNP per identified locus was annotated with Combined Annotation Dependent Depletion (CADD)^39^ scores, which predict how deleterious the SNP effect is on protein structure/function, and RegulomeDB^40^ scores, which predict the likelihood of regulatory functionality of SNPs. To investigate previous phenotype associations, the identified loci were queried in the GWAS catalog.^41^ Loci were also queried in the GTEx portal (GTEx v8)^42^ for known expression quantitative trait loci (eQTLs) across multiple tissues. Identified lead variants were mapped to genes using the Open Targets Genetics platform (https://genetics.opentargets.org/)^43^ that provides a Variant to Gene (V2G) association score for each variant-gene prediction, to assign likely causal genes for a given variant. For each lead variant, we considered the top three genes with the highest V2G score as well as the closest gene in case it was not among the top three genes with the highest V2G score. To identify drug-gene interactions, mapped genes were queried in the drug-gene interaction database (dgidb) v5.0.8 (06/12/2024).^44^ More details can be found in Supplementary Methods S1.

### Mendelian randomization and colocalization analyses

To estimate the potential causal relationship of TRS on smoking initiation as well as of smoking initiation on TRS, we used MR (R version 4.1.1, TwoSampleMR version 0.5.6)^45^ and reported results for inverse variance weighted,^46^ weighted median,^47^ weighted mode,^48^ and MR Egger^49^ (Supplementary Methods S1). Since TRS did not have any genome-wide significant SNPs (*P* < 5e-08), we used a reduced threshold for SNP inclusion (*P* < 1e-05) when TRS was set as the exposure. This is consistent with previous MR studies with GWAS of low statistical power.^50^^,^^51^ In addition, when TRS was set as the exposure, we performed pleiotropy and heterogeneity tests, including a leave-one-SNP-out analysis. For each locus identified through conjFDR, we performed a colocalization analysis (R version 4.1.1, coloc version 5.3.2).

### Polygenic prediction of TRS

Using PRSice-2,^52^ polygenic scores (PGSs) were calculated using summary statistics for TRS^3^ and smoking initiation.^24^ We compared PGS based on standard GWAS-ranked lead SNPs with PGS based on condFDR-based ranking, applying the pleioPGS approach,^53^ using variant effect sizes derived from the original TRS GWAS. We calculated the PGSs for specific numbers of lead SNPs rather than for significance thresholds to allow for direct comparison between the approaches, at equal numbers of SNPs in each set. We compared the top 100 (approximate number of SNPs *P* < 1e^-5^ in the original TRS GWAS) to 105 000 SNPs (number of independent SNPs *P* < 1 in the original TRS GWAS). Sex, age and the first 10 genetic principal components were included as covariates. The variance in TRS explained by the 3 different PGSs (TRS, smoking initiation, cFDR-based) was evaluated.

The target sample includes 1635 individuals (819 TRS and 816 non-TRS) from the therapeutic drug monitoring (TDM) service at the Center for Psychopharmacology in Diakonhjemmet Hospital, Oslo, Norway, between January 2005 and August 2022. TRS was defined based on the use/prescription of clozapine, the main drug indicated in TRS,^54^ verified by detectable serum concentrations of clozapine. Non-TRS patients had no history of clozapine treatment and had only used other antipsychotics (according to TDM records). Of the 1635 individuals, 912 were males (481 TRS and 431 non-TRS), and 723 were females (338 TRS and 385 non-TRS). The Regional Committee for Medical and Health Research Ethics and the Investigational Review Board at Diakonhjemmet Hospital approved the study. More information as well as information about genotyping and imputation, can be found in Supplementary Methods S1.

### Genetically informed drug prioritization

Genes identified from Open Targets Genetics^43^ were studied within networks of protein–protein interactions (PPIs) of gene products, using the latest version of the human protein interactome,^55^ consisting of 18 217 unique proteins (nodes) interconnected by 329 506 PPIs after removing self-loops. As most approved drugs do not target disease-associated proteins but bind to proteins in their network vicinity,^56^ we defined a network not only including the genes identified from Open Targets Genetics,^43^ but also genes in their immediate network proximity. To define a TRS network, we used the method network propagation,^57–59^ implemented in the Cytoscape^60^ application Diffusion.^59^ Genes identified from Open Targets Genetics^43^ were used as input query genes, and the top 1% of proteins from the diffusion output were included in the TRS network. The Drug Gene Interaction Database (DGIdb, (https://www.dgidb.org/) v.5.0.8^44^ was used to identify drug-gene interactions between approved drugs and genes in the TRS network. Gene-set enrichment analysis (GSEA) was performed to test for enrichment of drug-gene interactions within the TRS network (more details in Supplementary Methods S1).

## RESULTS

### Genetic overlap between TRS and smoking initiation

**Figure 1:**
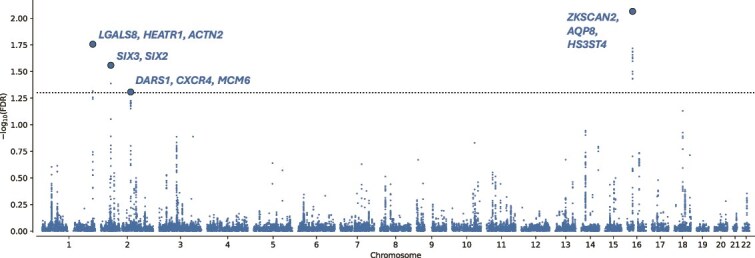
Manhattan plot showing genetic variants associated with treatment-resistant schizophrenia and smoking initiation by conjunctional false discovery rate (conjFDR) analysis, conjFDR <0.05. The y-axis represents the -log10 transformed conjFDR values for each SNP, and the x-axis represents chromosomal positions. The dotted horizontal line indicates the threshold for significant shared associations (conjFDR <0.05). Blue circles over the threshold represent lead SNPs with their respective top three genes with the highest variant-to-gene scores.

Genetic correlation analyses showed a significant positive correlation between TRS and smoking initiation (r_g_ = 0.4716, SE = 0.1277, z-score = 3.6921, *P*-value = .0002), also when TRS was conditioned on schizophrenia (r_g_ = 0.4729, SE = 0.1275, z-score = 3.7102, *P*-value = .0002). The conditional QQ-plots indicate cross-trait polygenic enrichment between TRS and smoking initiation (Figure S1). This is demonstrated by the leftward deflection, showing an increase in associations with TRS as a function of significance in smoking initiation. At conjFDR <0.05, we identified four loci jointly associated with TRS and smoking initiation (Table 1, Figure 1). The same four loci were identified at condFDR <0.05, with the only difference that the locus on chromosome 16 had another lead SNP (rs9928337). A list of all candidate variants in the identified loci is provided in Table S1 (condFDR) and Table S2 (conjFDR). While two of the identified loci (both on chromosome 2, lead SNP rs494904 and rs4076010) had a positive direction of effect in the TRS GWAS and the smoking initiation GWAS (increased risk for both TRS and smoking initiation), the locus on chromosome 1 (lead SNP rs12030126) and the locus on chromosome 16 (lead SNP rs9935028) had opposite direction of effect (decreased risk for TRS and increased risk for smoking initiation). Investigation of the four identified loci in the GWAS catalog^41^ showed that one locus (lead SNP rs494904) has been previously associated with both alcohol use disorder and problematic alcohol use,^61^ while no associations have been reported for the other identified loci.

**Table 1:** Novel loci for treatment-resistant schizophrenia (TRS) identified through conditional and conjunctional false discovery rate analysis with smoking initiation.

Lead SNP	Locus (chr: start-end)	A1/A2 ^a^	Mapped genes	*P*-value in TRS GWAS	Beta (SE) in TRS GWAS	*P*-value in smoking GWAS	Beta (SE) in smoking GWAS	conjFDR
**rs12030126**	1: 236808558 - 236 854 973	G/T	*LGALS8, HEATR1*, ***ACTN2***	3.51e^-5^	−0.112 (0.027)	2.14e^-5^	0.007 (0.002)	0.01752
**rs494904**	2: 45130410 - 45 157 336	C/T	***SIX3***, *SIX2*	5.77e^-5^	0.100 (0.025)	7.63e^-11^	0.010 (0.002)	0.02762
**rs4076010**	2: 137066601 - 137 119 376	T/C	*DARS1*, ***CXCR4***, *MCM6*	1.08e^-4^	0.090 (0.023)	2.28e^-3^	0.004 (0.002)	0.04924
**rs9935028 (rs9928337)**	16: 25382373 - 25 454 780	G/A	***ZKSCAN2***, *AQP8, HS3ST4*, (*LCMT1*)	3.56e^-6^	−0.104 (0.022)	9.07e^-4^	0.005 (0.002)	0.00861

Lead SNPs in independent genomic loci jointly associated with TRS and smoking initiation at conditional/conjunctional false discovery rate threshold <0.05. Genomic regions separated by less than 250 kb were merged into a single locus. The table lists chromosomal positions (chr) and includes the top three mapped genes identified through Open Targets Genetics.^43^ The nearest gene to the index SNP (in GRCh37 coordinates) is in bold font. The gene *LCMT1* was among the top three mapped genes to the lead SNP identified in condFDR (rs9928337).

^a^A1 is the effect allele and A2 is the reference allele.

Functional annotations for these loci do not suggest the lead SNPs to be deleterious (CADD scores <12.37)^39^ or likely to have regulatory functionality (RegulomeDB scores = 5-7).^40^ Assessment of the variant-gene relationships in the GTEx database^42^ showed significant associations between the lead SNP rs12030126 on chromosome 1 and *LGALS8* gene expression, with most significant associations in adipose tissue (*P* = 4.2e^-7^), the anterior cingulate cortex (*P* = 1.7e^-5^), and nucleus accumbens (*P* = 5.4e^-5^). The SNP was also associated with *HEATR1* gene expression, which was most significant in the esophagus (2.7e^-12^) and cerebellum (*P* = 6.6e^-11^), as well as with expression of *ACTN2* in esophagus (2.8e^-19^). The lead SNP rs9935028 on chromosome 16 was associated with expression of *ZKSCAN2* in muscle (*P* = 2.5e^-9^) and adipose tissue (*P* = 7.0e^-5^). Further assessment of the mapped genes in the DGIdb^44^ showed that *AQP8* interacts with 7 approved drugs (Table S3), most of which are prostaglandin analogs or derivatives indicated for the treatment of hypertension and/or hormonal or reproductive functions. Moreover, *CXCR4* interacts with 5 approved drugs primarily indicated for cancer treatment, and *ACTN2* interacts with adenosine triphosphate (Table S3). No interactions with approved drugs were identified for the other genes.

### Mendelian randomization and colocalization

Although the GWAS of TRS lacked power to estimate causal effects on smoking initiation using genome-wide significant loci, a relaxed threshold (*P* < 1e^-5^) showed a putative causal link to smoking initiation (*P* < .05) using the inverse variance weighted, weighted median, and weighted mode methods (Table S4). No evidence of a causal effect of smoking on TRS was seen (Table S4). The instrument (SNP) level (*N* = 15) and inverse variance weighted F-statistics were all >10, suggesting a lack of weak instrumentation of TRS (Table S5). Leave-one-SNP out analyses for the TRS SNPs showed consistency in SNP effects and *P*-values across runs, suggesting a stable association and likely no biases due to any single SNP (Table S6). Results from the heterogeneity tests showed a non-significant Q-statistic but some indication of heterogeneity based on I-squared (Q = 9.97, *P* = .76, I^2^ = 0.96). There was no sign of horizontal pleiotropy based on the Egger intercept (Egger intercept: -0.0008, SE = 0.0026, *P* = .7562). For the locus on chromosome 2 (lead SNP rs494904), the colocalization analysis indicates that the traits TRS and smoking initiation are associated and share a causal variant in this region (94% probability). There was no clear evidence for colocalization for the other loci (Table S7).

### Polygenic prediction of TRS

**Figure 2:**
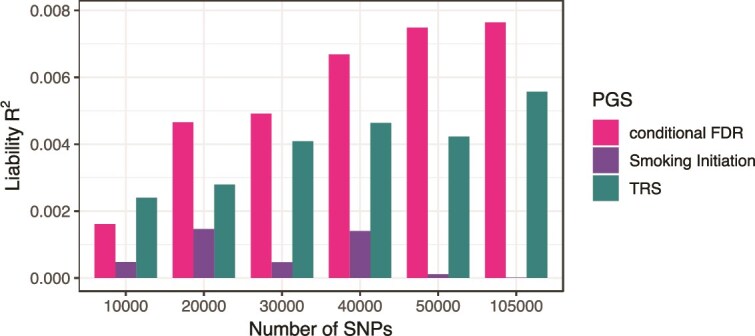
Explained variance (on the liability scale) in treatment-resistant schizophrenia (TRS) for polygenic scores (PGSs) based on three different SNP rankings: TRS (green), smoking initiation (purple), and conditional false discovery rate-based ranking of TRS conditioned on smoking initiation (pink). PGS were constructed for top 100-105 000 independent SNPs from the TRS GWAS summary statistics. The results of explained variance for all SNP ranges are presented in Figure S2.

We compared the top 100-105 000 SNPs using original TRS GWAS *P*-value ranking and condFDR-based ranking (Figure S1), hypothesizing that the boosted power from our conditional analysis would select more informative variants than standard GWAS, resulting in improved PGS performance. The PGS based on condFDR-ranked SNPs explained more of the variance in TRS compared to both the standard TRS PGS and the smoking initiation PGS (Figure S1), with the highest variance explained (R^2^ = 0.0079, *P* = .002) at 52000 SNPs. The highest variance explained by the TRS PGS was at 65000 SNPs (R^2^ = 0.0065, *P* = .005), while condFDR-based ranking achieved equivalent predictive performance by using almost half the number of SNPs (R^2^ = 0.0066 at 37000 SNPs), which indicates a more efficient capture of relevant genetic signal with condFDR-based ranking. Figure 2 shows the PGS performance in TRS for the PGS based on condFDR-ranked SNPs and the standard TRS PGS at 10000, 20000, 30 000, 40 000, 50 000, and 105 000 SNPs. The smoking initiation PGS is also plotted for comparison.

### Genetically informed drug prioritization

**Figure 3:**
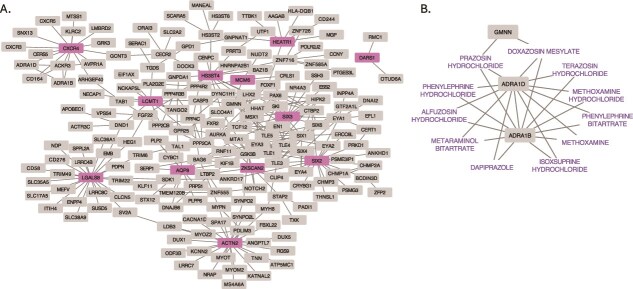
Treatment-resistant schizophrenia network (A) and drug target genes in the network that were enriched for alpha-1 adrenergic receptor modulators (B). The input genes to build the network are highlighted in pink. Nodes refer to genes or drugs, and edges refer to gene-drug interactions or gene–gene interactions through identified protein–protein interactions between gene products (proteins).

The genes included in the TRS network (*N* = 194) and the corresponding diffusion output values can be found in Table S8. Drug target genes in the TRS network were enriched (*P* < .05) for targets of 11 drugs, most of which were alpha-1 adrenergic receptor agonists/antagonists, used to manage cardiovascular conditions. However, after correcting for the total number of drug-gene interactions (*N* = 25 290), none of the enrichments remained significant (FDR > 0.05) (Table S9). The TRS network and the drugs identified based on GSEA are shown in Figure 3.

## Discussion

Applying the condFDR framework,^1^^,^^34^^,^^35^ we increased discovery in an underpowered GWAS by leveraging genetic overlap with a second, well-powered GWAS.^62^^,^^63^ We identified four novel loci for TRS, while no loci were identified in the original TRS GWAS.^3^ Some of the mapped genes have been previously associated with schizophrenia. *LGALS8* (mapped to rs12030126 on chromosome 1), encoding galectin-8, is downregulated in the hippocampus of schizophrenia patients.^64^ It has also been linked to cigarette smoking, with evidence suggesting that cigarette smoke-induced autophagy impairment leads to increased galectin-8 and inflammation.^65^ Suppressing *LGALS8* activity has been shown to influence inflammatory pathways and mitigate tissue damage by reducing inflammatory cell recruitment and activation, leading to decreased inflammation and tissue protection. As chronic low-grade inflammation is a key factor in TRS,^66^ immune-modulating treatment options have been suggested for TRS.^66^^,^^67^ While *ZKSCAN2* (mapped to rs9935028 on chromosome 16) has not been previously associated with schizophrenia, other members of the zinc finger transcription factors family (eg, *ZKSCAN3* and *ZKSCAN4*) have shown associations with gray matter reduction in schizophrenia.^68^^,^^69^ In addition, some evidence suggests that zinc finger proteins may be linked to response to psychotropic medications, but the functions of the investigated proteins are largely uncharacterized, thus further studies are needed to elucidate the role of zinc finger proteins in TRS.^70^ Aquaporins, such as *AQP3* and *AQP4*, have been associated with the pathophysiology of schizophrenia.^71^^,^^72^ While *AQP8* (mapped to rs9935028 on chromosome 16) is expressed in the brain, its function in the CNS remains unclear.^73^ AQP8 facilitates the transmembrane transport of hydrogen peroxide, thereby regulating intracellular levels of reactive oxygen species and inflammatory responses. Under proinflammatory conditions, such as in TRS, altered AQP8 may cause mitochondrial dysfunction.^74^ Alpha-actinin-2, encoded by *ACTN2* (mapped to the locus on chromosome 1, rs12030126), could indirectly associate with schizophrenia and possibly TRS through its role in interacting with glutamate N-methyl-D-aspartate (NMDA) receptors,^75^ which have been linked to the pathophysiology of both schizophrenia and TRS.^76^ The chemokine receptor type 4 (*CXCR4*, mapped to rs4076010 on chromosome 2) has also shown interactions with NMDA receptors, leading to reduced NMDA receptor signaling that has been associated with the pathophysiology of schizophrenia.^77^ Finally, mutations in the *SIX3* gene (mapped to rs494904 on chromosome 2) have been associated with both schizophrenia^78^ and smoking.^79^ In addition, the 6 family of transcription factors, especially SIX4, is associated with promoting inflammatory pathways.^80^ Mutations in the *SIX4* gene could therefore be related to the persistent inflammation seen in TRS. However, an association between the lead SNP (rs494904) and alcohol use disorder and problematic alcohol consumption^61^ could be a confounder of the shared genetic signal between TRS and smoking initiation, because alcohol comorbidity is not captured in the TRS GWAS. However, larger GWASs of TRS are still needed, and the genes mapped to the identified loci should be further investigated in functional studies to validate if these genes are likely causal for TRS.

Several studies have shown significant genetic correlations between schizophrenia and smoking initiation (r_g_ ranging between 0.10 and 0.16),^24^^,^^81^^,^^82^ indicating a shared genetic basis. In a GWAS of smoking behaviors among schizophrenia cases, it was demonstrated that a PGS for smoking initiation was partially shared between schizophrenia cases and the general population.^81^ However, the molecular mechanisms underlying the schizophrenia-smoking association are not completely understood.^81^ While a genetic overlap between schizophrenia and smoking behavior has been demonstrated in studies applying the cond/conj FDR method,^83^^,^^84^ it remains unknown if shared genetic factors with smoking initiation are different among TRS and non-TRS patients. We demonstrate a significant positive genetic correlation between TRS and smoking initiation that is significantly higher than the genetic correlation between smoking initiation and schizophrenia, supporting a shared genetic basis between TRS and smoking initiation that is independent from the genetic contribution of schizophrenia. By leveraging the identified genetic overlap between TRS and smoking initiation, we show that a PGS based on condFDR-ranked SNPs explained the greatest variance in TRS compared to both the standard TRS PGS and the smoking initiation PGS, albeit the predicted value was small. This pleioPGS approach outperformed the standard GWAS-based ranking, utilizing less SNPs in the PGS, despite using the same SNP weightings, indicating a more efficient capture of relevant genetic signal with condFDR-based ranking. While none of the PGSs provide any clinically useful predictions of TRS, we demonstrate that the secondary trait (smoking initiation) has relevant genetic overlap with the primary trait (TRS) for boosting prediction of TRS, with the pleioPGS approach improving PGS performance, which may become useful in the future once larger TRS GWASs become available.

The network analyses implicated two alpha-1-adrenergic receptors (ADRA1B and ADRA1D), and we shortlist several drugs acting on these receptors. Results from several clinical trials have demonstrated that alpha-1-adrenergic receptor antagonists are efficacious in the treatment of negative symptoms and social functioning deficits in schizophrenia.^85–87^ Clozapine, which shows superior efficacy compared to conventional antipsychotic drugs, shows significant affinity for alpha-adrenergic receptors, especially alpha-1-adrenergic receptors.^88^ Of note, clozapine exerts an advantageous therapeutic effect on negative and cognitive symptoms, which are usually rather resistant to treatment with other antipsychotics.^89^ Because clozapine displays significant affinities for several neurotransmitter receptors, including muscarinic, histaminergic, and adrenergic receptors, with comparatively low D_2_ dopamine receptor binding,^88^^,^^90^ a critical question is which of these receptor affinities may contribute to clozapine’s superior therapeutic effect.^90^^,^^91^ It has been hypothesized that clozapine’s superior efficacy is related to its alpha-adrenoceptor modulation, stating that alpha-1- and alpha-2-adrenoceptor blocking stabilizes the dysregulated central dopaminergic systems in schizophrenia.^91^ Although the mechanisms involved remain to be fully understood, it has been suggested that antagonism of alpha-1-adrenergic receptors may suppress striatal hyperdopaminergia involved in positive symptoms, while alpha-2-adrenergic receptor antagonism may improve prefrontal dopaminergic functioning, thereby reducing negative and cognitive symptoms.^91^ Moreover, alpha-1-adrenoceptor antagonism has been related to the metabolic side effects of antipsychotics,^88^^,^^92^ with clozapine being associated with the largest degree of metabolic dysfunction.^93^ Interestingly, it has been suggested that clozapine’s effectiveness may be related to its metabolic side effects.^62^^,^^93^ Taken together, these results indicate that alpha-adrenergic receptor antagonism may be considered as treatment for TRS after further investigation.

In conclusion, by conditioning on smoking initiation, we boosted discovery and identified four novel loci associated with TRS. These findings suggest that shared genetic mechanisms influence TRS and smoking behavior. In addition, the results indicate that alpha-1-adrenergic receptors may be involved in the pathobiology of TRS and might possibly being related to the superior efficacy of clozapine. Together, our results provide new insights into the biological underpinnings of TRS.

## Supplementary Material

Supplementary_material_pyag035

## Data Availability

The code for cond/conjFDR and pleioPGS is publicly available at https://github.com/precimed/pleiofdr and https://github.com/norment/open-science/tree/main/2021_VanderMeer_medRxiv_pleioPGS, respectively.

## References

[1] Smeland OB, Frei O, Shadrin A, et al. Discovery of shared genomic loci using the conditional false discovery rate approach. *Hum Genet*. 2020;139:85–94. 10.1007/s00439-019-02060-2 31520123

[2] Koch E, Smart S, Einarsson G, et al. Recommendations for defining treatment outcomes in major psychiatric disorders using real-world data. *Lancet Psychiatry*. 2025;12:457–468. 10.1016/S2215-0366(25)00061-6 40222385

[3] Pardinas AF, Smart SE, Willcocks IR, et al. Interaction testing and polygenic risk scoring to estimate the Association of Common Genetic Variants with treatment resistance in schizophrenia. *JAMA Psychiatry*. 2022;79:260–269. 10.1001/jamapsychiatry.2021.3799 35019943 PMC8756361

[4] Taipale H, Puranen A, Mittendorfer-Rutz E, et al. Antipsychotic use among persons with schizophrenia in Sweden and Finland, trends and differences. *Nord J Psychiatry*. 2021;75:315–322. 10.1080/08039488.2020.1854853 33331804

[5] Wellesley Wesley E, Patel I, Kadra-Scalzo G, et al. Gender disparities in clozapine prescription in a cohort of treatment-resistant schizophrenia in the South London and Maudsley case register. *Schizophr Res*. 2021;232:68–76. 10.1016/j.schres.2021.05.006 34022618

[6] de Freitas DF, Patel I, Kadra-Scalzo G, et al. Ethnic inequalities in clozapine use among people with treatment-resistant schizophrenia: a retrospective cohort study using data from electronic clinical records. *Soc Psychiatry Psychiatr Epidemiol*. 2022;57:1341–1355. 10.1007/s00127-022-02257-3 35246709 PMC9246775

[7] Kyllesø L, Smith RL, Karlstad Ø, Andreassen OA, Molden E. Undetectable or subtherapeutic serum levels of antipsychotic drugs preceding switch to clozapine. *NPJ Schizophr*. 2020;6:17. 10.1038/s41537-020-0107-7 32681055 PMC7367852

[8] Lenk H, Smith RL, O'Connell KS, Andreassen OA, Molden E. Rapid metabolism underlying subtherapeutic serum levels of atypical antipsychotics preceding clozapine treatment: a retrospective analysis of real-world data. *CNS Drugs*. 2024;38:473–480. 10.1007/s40263-024-01079-y 38635089 PMC11098931

[9] Kane JM, Correll CU. The role of clozapine in treatment-resistant schizophrenia. *JAMA Psychiatry*. 2016;73:187–188. 10.1001/jamapsychiatry.2015.2966 26841681

[10] Kowalec K, Lu Y, Sariaslan A, et al. Increased schizophrenia family history burden and reduced premorbid IQ in treatment-resistant schizophrenia: a Swedish National Register and genomic study. *Mol Psychiatry*. 2021;26:4487–4495. 10.1038/s41380-019-0575-1 31712719 PMC9731609

[11] Tiihonen J, Mittendorfer-Rutz E, Majak M, et al. Real-world effectiveness of antipsychotic treatments in a Nationwide cohort of 29 823 patients with schizophrenia. *JAMA Psychiatry*. 2017;74:686–693. 10.1001/jamapsychiatry.2017.1322 28593216 PMC5710250

[12] Huhn M, Nikolakopoulou A, Schneider-Thoma J, et al. Comparative efficacy and tolerability of 32 oral antipsychotics for the acute treatment of adults with multi-episode schizophrenia: a systematic review and network meta-analysis. *Lancet*. 2019;394:939–951. 10.1016/S0140-6736(19)31135-3 31303314 PMC6891890

[13] Wagner E, Siafis S, Fernando P, et al. Efficacy and safety of clozapine in psychotic disorders-a systematic quantitative meta-review. *Transl Psychiatry*. 2021;11:487. 34552059 10.1038/s41398-021-01613-2 PMC8458455

[14] Myles N, Myles H, Xia S, et al. Meta-analysis examining the epidemiology of clozapine-associated neutropenia. *Acta Psychiatr Scand*. 2018;138:101–109. 10.1111/acps.12898 29786829

[15] Oloyede E, Blackman G, Whiskey E, et al. Clozapine haematological monitoring for neutropenia: a global perspective. *Epidemiol Psychiatr Sci*. 2022;31:e83. 10.1017/S204579602200066X 36426600 PMC9714212

[16] Kumra S, Kranzler H, Gerbino-Rosen G, et al. Clozapine and “high-dose” olanzapine in refractory early-onset schizophrenia: a 12-week randomized and double-blind comparison. *Biol Psychiatry*. 2008;63:524–529. 10.1016/j.biopsych.2007.04.043 17651705

[17] Conley RR, Kelly DL. Management of treatment resistance in schizophrenia. *Biol Psychiatry*. 2001;50:898–911. 10.1016/S0006-3223(01)01271-9 11743944

[18] Kinon BJ . The Group of Treatment Resistant Schizophrenias. Heterogeneity in treatment resistant schizophrenia (TRS). *Front*. *Psychiatry*. 2018;9:757. 10.3389/fpsyt.2018.00757 PMC6363683 30761026

[19] Trubetskoy V, Pardinas AF, Qi T, et al. Mapping genomic loci implicates genes and synaptic biology in schizophrenia. *Nature*. 2022;604:502–508. 10.1038/s41586-022-04434-5 35396580 PMC9392466

[20] Andreassen OA, Hindley G, Frei O, Smeland OB. New insights from the last decade of research in psychiatric genetics- discoveries, challenges and clinical implications. *World Psychiatry*. 2023;22:4–24. 10.1002/wps.21034 36640404 PMC9840515

[21] Lenk HC, Koch E, O'Connell KS, et al. Genome-wide association analysis of treatment resistant schizophrenia for variant discovery and polygenic assessment. *Hum Genomics*. 2024;18:108. 39334510 10.1186/s40246-024-00673-x PMC11438281

[22] Pardinas AF, Owen MJ, Walters JTR. Pharmacogenomics: a road ahead for precision medicine in psychiatry. *Neuron*. 2021;109:3914–3929. 10.1016/j.neuron.2021.09.011 34619094

[23] Ding JB, Hu K. Cigarette smoking and schizophrenia: etiology, clinical, pharmacological, and treatment implications. *Schizophr Res Treatment*. 2021;2021:7698030–7698038. 34938579 10.1155/2021/7698030 PMC8687814

[24] Liu M, Jiang Y, Wedow R, et al. Association studies of up to 1.2 million individuals yield new insights into the genetic etiology of tobacco and alcohol use. *Nat Genet*. 2019;51:237–244. 10.1038/s41588-018-0307-5 30643251 PMC6358542

[25] Wootton RE, Richmond RC, Stuijfzand BG, et al. Evidence for causal effects of lifetime smoking on risk for depression and schizophrenia: a mendelian randomisation study. *Psychol Med*. 2020;50:2435–2443. 10.1017/S0033291719002678 31689377 PMC7610182

[26] Dalack GW, Healy DJ, Meador-Woodruff JH. Nicotine dependence in schizophrenia: clinical phenomena and laboratory findings. *Am J Psychiatry*. 1998;155:1490–1501. 10.1176/ajp.155.11.1490 9812108

[27] Parikh V, Kutlu MG, Gould TJ. nAChR dysfunction as a common substrate for schizophrenia and comorbid nicotine addiction: current trends and perspectives. *Schizophr Res*. 2016;171:1–15. 10.1016/j.schres.2016.01.020 26803692 PMC4762752

[28] Osimo EF, Perry BI, Mallikarjun P, et al. Predicting treatment resistance from first-episode psychosis using routinely collected clinical information. *Nat Ment Health*. 2023;1:25–35. 10.1038/s44220-022-00001-z 37034013 PMC7614410

[29] Iasevoli F, Balletta R, Gilardi V, Giordano S, de Bartolomeis A. Tobacco smoking in treatment-resistant schizophrenia patients is associated with impaired cognitive functioning, more severe negative symptoms, and poorer social adjustment. *Neuropsychiatr Dis Treat*. 2013;9:1113–1120. 23950651 10.2147/NDT.S47571 PMC3742345

[30] Barrangou-Poueys-Darlas M, Guerlais M, Laforgue EJ, et al. CYP1A2 and tobacco interaction: a major pharmacokinetic challenge during smoking cessation. *Drug Metab Rev*. 2021;53:30–44. 10.1080/03602532.2020.1859528 33325257

[31] Beunk L, Nijenhuis M, Soree B, et al. Dutch pharmacogenetics working group (DPWG) guideline for the gene-drug interaction between CYP2D6, CYP3A4 and CYP1A2 and antipsychotics. *Eur J Hum Genet*. 2024;32:278–285. 10.1038/s41431-023-01347-3 37002327 PMC10923774

[32] Scherf-Clavel M, Samanski L, Hommers LG, Deckert J, Menke A, Unterecker S. Analysis of smoking behavior on the pharmacokinetics of antidepressants and antipsychotics: evidence for the role of alternative pathways apart from CYP1A2. *Int Clin Psychopharmacol*. 2019;34:93–100. 10.1097/YIC.0000000000000250 30557209

[33] Djordjevic N, Radmanovic B, Cukic J, et al. Cigarette smoking and heavy coffee consumption affecting response to olanzapine: the role of genetic polymorphism. *World J Biol Psychiatry*. 2020;21:29–52. 10.1080/15622975.2018.1548779 30513034

[34] Andreassen OA, Thompson WK, Dale AM. Boosting the power of schizophrenia genetics by leveraging new statistical tools. *Schizophr Bull*. 2014;40:13–17. 10.1093/schbul/sbt168 24319118 PMC3885310

[35] Andreassen OA, Djurovic S, Thompson WK, et al. Improved detection of common variants associated with schizophrenia by leveraging pleiotropy with cardiovascular-disease risk factors. *Am J Hum Genet*. 2013;92:197–209. 10.1016/j.ajhg.2013.01.001 23375658 PMC3567279

[36] Bulik-Sullivan BK, Loh PR, Finucane HK, et al. LD score regression distinguishes confounding from polygenicity in genome-wide association studies. *Nat Genet*. 2015;47:291–295. 10.1038/ng.3211 25642630 PMC4495769

[37] Zhu Z, Zheng Z, Zhang F, et al. Causal associations between risk factors and common diseases inferred from GWAS summary data. *Nat Commun*. 2018;9:224. 10.1038/s41467-017-02317-2 29335400 PMC5768719

[38] Watanabe K, Taskesen E, van Bochoven A, Posthuma D. Functional mapping and annotation of genetic associations with FUMA. *Nat Commun*. 2017;8:1826. 10.1038/s41467-017-01261-5 29184056 PMC5705698

[39] Kircher M, Witten DM, Jain P, O'Roak BJ, Cooper GM, Shendure J. A general framework for estimating the relative pathogenicity of human genetic variants. *Nat Genet*. 2014;46:310–315. 10.1038/ng.2892 24487276 PMC3992975

[40] Boyle AP, Hong EL, Hariharan M, et al. Annotation of functional variation in personal genomes using RegulomeDB. *Genome Res*. 2012;22:1790–1797. 10.1101/gr.137323.112 22955989 PMC3431494

[41] Buniello A, MacArthur JAL, Cerezo M, et al. The NHGRI-EBI GWAS Catalog of published genome-wide association studies, targeted arrays and summary statistics 2019. *Nucleic Acids Res*. 2019;47:D1005–D1012. 10.1093/nar/gky1120 30445434 PMC6323933

[42] The GTEx Consortium . Human genomics. The genotype-tissue expression (GTEx) pilot analysis: multitissue gene regulation in humans. *Science*. 2015;348:648–660. 10.1126/science.1262110 25954001 PMC4547484

[43] Ghoussaini M, Mountjoy E, Carmona M, et al. Open targets genetics: systematic identification of trait-associated genes using large-scale genetics and functional genomics. *Nucleic Acids Res*. 2021;49:D1311–D1320. 10.1093/nar/gkaa840 33045747 PMC7778936

[44] Cannon M, Stevenson J, Stahl K, et al. DGIdb 5.0: rebuilding the drug-gene interaction database for precision medicine and drug discovery platforms. *Nucleic Acids Res*. 2024;52:D1227–D1235. 10.1093/nar/gkad1040 37953380 PMC10767982

[45] Hemani G, Zheng J, Elsworth B, et al. The MR-base platform supports systematic causal inference across the human phenome. *eLife*. 2018;7:1-29. 10.7554/eLife.34408 PMC5976434 29846171

[46] Burgess S, Butterworth A, Thompson SG. Mendelian randomization analysis with multiple genetic variants using summarized data. *Genet Epidemiol*. 2013;37:658–665. 10.1002/gepi.21758 24114802 PMC4377079

[47] Bowden J, Davey Smith G, Haycock PC, Burgess S. Consistent estimation in mendelian randomization with some invalid instruments using a weighted median estimator. *Genet Epidemiol*. 2016;40:304–314. 10.1002/gepi.21965 27061298 PMC4849733

[48] Hartwig FP, Davey Smith G, Bowden J. Robust inference in summary data mendelian randomization via the zero modal pleiotropy assumption. *Int J Epidemiol*. 2017;46:1985–1998. 10.1093/ije/dyx102 29040600 PMC5837715

[49] Bowden J, Davey Smith G, Burgess S. Mendelian randomization with invalid instruments: effect estimation and bias detection through egger regression. *Int J Epidemiol*. 2015;44:512–525. 10.1093/ije/dyv080 26050253 PMC4469799

[50] Cheng W, Parker N, Karadag N, et al. The relationship between cannabis use, schizophrenia, and bipolar disorder: a genetically informed study. *Lancet Psychiatry*. 2023;10:441–451. 10.1016/S2215-0366(23)00143-8 37208114 PMC10311008

[51] Pasman JA, Verweij KJH, Gerring Z, et al. GWAS of lifetime cannabis use reveals new risk loci, genetic overlap with psychiatric traits, and a causal influence of schizophrenia. *Nat Neurosci*. 2018;21:1161–1170. 10.1038/s41593-018-0206-1 30150663 PMC6386176

[52] Choi SW, O'Reilly PF. PRSice-2: polygenic risk score software for biobank-scale data. *Gigascience*. 2019;8:1–6. 10.1093/gigascience/giz082 PMC6629542 31307061

[53] van der Meer D, Shadrin AA, O’Connell K, et al. Boosting schizophrenia genetics by utilizing genetic overlap with brain morphology. *Biol Psychiatry*. 2022;92:291–298. 10.1016/j.biopsych.2021.12.007 35164939 PMC12012303

[54] FDA . U.S. Food and Drug Administration, Clozaril, Label Approval, NDA no. 019758 2014.

[55] Morselli Gysi D, Barabasi AL. Noncoding RNAs improve the predictive power of network medicine. *Proc Natl Acad Sci USA*. 2023;120:e2301342120. 10.1073/pnas.2301342120 37906646 PMC10636370

[56] Yildirim MA, Goh KI, Cusick ME, Barabasi AL, Vidal M. Drug-target network. *Nat Biotechnol*. 2007;25:1119–1126. 10.1038/nbt1338 17921997

[57] Köhler S, Bauer S, Horn D, Robinson PN. Walking the interactome for prioritization of candidate disease genes. *Am J Hum Genet*. 2008;82:949–958. 10.1016/j.ajhg.2008.02.013 18371930 PMC2427257

[58] Vanunu O, Magger O, Ruppin E, Shlomi T, Sharan R. Associating genes and protein complexes with disease via network propagation. *PLoS Comput Biol*. 2010;6:e1000641. 10.1371/journal.pcbi.1000641 20090828 PMC2797085

[59] Carlin DE, Demchak B, Pratt D, Sage E, Ideker T. Network propagation in the cytoscape cyberinfrastructure. *PLoS Comput Biol*. 2017;13:e1005598. 10.1371/journal.pcbi.1005598 29023449 PMC5638226

[60] Shannon P, Markiel A, Ozier O, et al. Cytoscape: a software environment for integrated models of biomolecular interaction networks. *Genome Res*. 2003;13:2498–2504. 10.1101/gr.1239303 14597658 PMC403769

[61] Zhou H, Sealock JM, Sanchez-Roige S, et al. Genome-wide meta-analysis of problematic alcohol use in 435,563 individuals yields insights into biology and relationships with other traits. *Nat Neurosci*. 2020;23:809–818. 10.1038/s41593-020-0643-5 32451486 PMC7485556

[62] O'Connell KS, Koch E, Lenk HC, et al. Polygenic overlap with body-mass index improves prediction of treatment-resistant schizophrenia. *Psychiatry Res*. 2023;325:115217. 10.1016/j.psychres.2023.115217 37146461 PMC10788293

[63] Koch E, Shadrin AA, Parker N, et al. Polygenic overlap with granulocyte counts identifies novel loci for clozapine metabolism and clozapine-induced agranulocytosis. *Neuropsychopharmacology*. 2025;50:947–955. 10.1038/s41386-025-02054-x 39827279 PMC12032044

[64] Petralia MC, Ciurleo R, Bramanti A, et al. Transcriptomic data analysis reveals a Down-expression of Galectin-8 in schizophrenia hippocampus. *Brain sciences*. 2021;11:973. 10.3390/brainsci11080973 34439592 PMC8392448

[65] Kono Y, Colley T, To M, et al. Cigarette smoke-induced impairment of autophagy in macrophages increases galectin-8 and inflammation. *Sci Rep*. 2021;11:335–313. 33432024 10.1038/s41598-020-79848-0 PMC7801483

[66] Leboyer M, Godin O, Terro E, et al. Immune signatures of treatment-resistant schizophrenia: a FondaMental academic Centers of expertise for schizophrenia (FACE-SZ) study. *Schizophr Bull Open*. 2021;2:sgab012. 10.1093/schizbullopen/sgab012 34901861 PMC8650073

[67] Sá ZC, Buinho A, Oliveira T, Soares FR, Rema JP, Novais F. Neuroinflamation treatment options in resistant schizophrenia—state of the art. *Curr Treat Options Psychiatry*. 2025;12:1-13. 10.1007/s40501-025-00356-x

[68] Chen J, Calhoun VD, Lin D, et al. Shared genetic risk of schizophrenia and gray matter reduction in 6p22.1. *Schizophr Bull*. 2019;45:222–232. 10.1093/schbul/sby010 29474680 PMC6293216

[69] Zhang Y, Lu T, Yan H, et al. Replication of association between schizophrenia and chromosome 6p21-6p22.1 polymorphisms in Chinese Han population. *PLoS One*. 2013;8:e56732–e56732. 10.1371/journal.pone.0056732 23437227 PMC3578928

[70] Squassina A, Meloni A, Chillotti C, Pisanu C. Zinc finger proteins in psychiatric disorders and response to psychotropic medications. *Psychiatr Genet*. 2019;29:132–141. 10.1097/YPG.0000000000000231 31464994

[71] Kunisawa K, Shimizu T, Kushima I, et al. Dysregulation of schizophrenia-related aquaporin 3 through disruption of paranode influences neuronal viability. *J Neurochem*. 2018;147:395–408. 10.1111/jnc.14553 30025158 PMC6205917

[72] Wu Y-F, Sytwu H-K, Lung F-W. Polymorphisms in the human aquaporin 4 gene are associated with schizophrenia in the southern Chinese Han population: a case-control study. *Front Psychiatry*. 2020;11:596–596. 10.3389/fpsyt.2020.00596 32676041 PMC7333661

[73] Rosu GC, Pirici I, Grigorie AA, et al. Distribution of aquaporins 1 and 4 in the central nervous system. *Curr Health Sci J*. 2019;45:218–226. 10.12865/CHSJ.45.02.14 31624651 PMC6778305

[74] Krüger C, Jörns A, Kaynert J, et al. The importance of aquaporin-8 for cytokine-mediated toxicity in rat insulin-producing cells. *Free Radic Biol Med*. 2021;174:135–143. 10.1016/j.freeradbiomed.2021.08.003 34363947

[75] Dunah AW, Wyszynski M, Martin DM, Sheng M, Standaert DG. Alpha-actinin-2 in rat striatum: localization and interaction with NMDA glutamate receptor subunits. *Brain Res Mol Brain Res*. 2000;79:77–87. 10.1016/S0169-328X(00)00102-9 10925145

[76] Okubo R, Okada M, Motomura E. Dysfunction of the NMDA receptor in the pathophysiology of schizophrenia and/or the Pathomechanisms of treatment-resistant schizophrenia. *Biomolecules*. 2024;14:1-21. 10.3390/biom14091128 PMC11430065 39334894

[77] Borroto-Escuela DO, Tarakanov AO, Bechter K, Fuxe K. IL1R2, CCR2, and CXCR4 may form heteroreceptor complexes with NMDAR and D2R: relevance for schizophrenia. *Front Psychiatry*. 2017;8:24. 10.3389/fpsyt.2017.00024 28261115 PMC5309215

[78] Li Y, Wang R, Qiao N, et al. Transcriptome analysis reveals determinant stages controlling human embryonic stem cell commitment to neuronal cells. *J Biol Chem*. 2017;292:19590–19604. 10.1074/jbc.M117.796383 28972157 PMC5712601

[79] Cheng Y, Dao C, Zhou H, et al. Multi-trait genome-wide association analyses leveraging alcohol use disorder findings identify novel loci for smoking behaviors in the million veteran program. *Transl Psychiatry*. 2023;13:148–149. 37147289 10.1038/s41398-023-02409-2 PMC10162964

[80] Zhang Z, Huang H, Peng L, et al. SIX4 activation in inflammatory response drives the transformation of colorectal epithelium into inflammation and tumor via feedback-enhancing inflammatory Signaling to induce tumor stemness Signaling. *Int J Biol Sci*. 2024;20:4618–4634. 10.7150/ijbs.93411 39309424 PMC11414381

[81] Peterson RE, Bigdeli TB, Ripke S, et al. Genome-wide analyses of smoking behaviors in schizophrenia: findings from the psychiatric genomics consortium. *J Psychiatr Res*. 2021;137:215–224. 10.1016/j.jpsychires.2021.02.027 33691233 PMC8096167

[82] Hartz SM, Horton AC, Hancock DB, et al. Genetic correlation between smoking behaviors and schizophrenia. *Schizophr Res*. 2018;194:86–90. 10.1016/j.schres.2017.02.022 28285025 PMC5811408

[83] Zuber V, Jönsson EG, Frei O, et al. Identification of shared genetic variants between schizophrenia and lung cancer. *Sci Rep*. 2018;8:674. 10.1038/s41598-017-16481-4 29330379 PMC5766533

[84] Rødevand L, Rahman Z, Hindley GFL, et al. Characterizing the shared genetic underpinnings of schizophrenia and cardiovascular disease risk factors. *Am J Psychiatry*. 2023;180:815–826. 10.1176/appi.ajp.20220660 37752828 PMC11780279

[85] Davidson M, Saoud J, Staner C, et al. Efficacy and safety of Roluperidone for the treatment of negative symptoms of schizophrenia. *Schizophr Bull*. 2022;48:609–619. 10.1093/schbul/sbac013 35211743 PMC9077422

[86] Rabinowitz J, Staner C, Saoud J, et al. Long-term effects of Roluperidone on negative symptoms of schizophrenia. *Schiz-ophr Res*. 2023;255:9–13. 10.1016/j.schres.2023.03.028 36933291

[87] James SH, Ahmed AO, Harvey PD, et al. Network intervention analysis indicates that roluperidone achieves its effect on negative symptoms of schizophrenia by targeting avolition. *Eur Neuropsychopharmacol*. 2024;87:18–23. 10.1016/j.euroneuro.2024.07.005 39024856

[88] Siafis S, Tzachanis D, Samara M, Papazisis G. Antipsychotic drugs: from receptor-binding profiles to metabolic side effects. *Curr Neuropharmacol*. 2018;16:1210–1223. 10.2174/1570159X15666170630163616 28676017 PMC6187748

[89] Meltzer HY . Role of serotonin in the action of atypical antipsychotic drugs. *Clin Neurosci*. 1995;3:64–75. 7583621

[90] de Bartolomeis A, Vellucci L, Barone A, et al. Clozapine’s multiple cellular mechanisms: what do we know after more than fifty years? A systematic review and critical assessment of translational mechanisms relevant for innovative strategies in treatment-resistant schizophrenia. *Pharmacol Ther*. 2022;236:1-41. 10.1016/j.pharmthera.2022.108236 35764175

[91] Svensson TH . Alpha-adrenoceptor modulation hypothesis of antipsychotic atypicality. *Prog Neuro-Psychopharmacol Biol Psychiatry*. 2003;27:1145–1158. 10.1016/j.pnpbp.2003.09.009 14642973

[92] Stanford SC, Heal DJ. Adrenoceptors: a focus on psychiatric disorders and their treatments. *Handb Exp Pharmacol*. 2024;285:507–554. 10.1007/164_2023_675 37495853

[93] Pillinger T, McCutcheon RA, Vano L, et al. Comparative effects of 18 antipsychotics on metabolic function in patients with schizophrenia, predictors of metabolic dysregulation, and association with psychopathology: a systematic review and network meta-analysis. *Lancet Psychiatry*. 2020;7:64–77. 10.1016/S2215-0366(19)30416-X 31860457 PMC7029416

